# Farmers’ willingness to pay for digital and conventional credit: Insight from a discrete choice experiment in Madagascar

**DOI:** 10.1371/journal.pone.0257909

**Published:** 2021-11-12

**Authors:** Yaw Sarfo, Oliver Musshoff, Ron Weber, Michael Danne

**Affiliations:** 1 Department for Agricultural Economics and Rural Development, Georg-August-Universität Göttingen, Göttingen, Germany; 2 Financial & Energy Sector Development, West Africa & Madagascar, KfW Bankengruppe, Frankfurt, Germany; Sveriges landbruksuniversitet - Campus Umea, SWEDEN

## Abstract

In recent decades, microfinance institutions with financial products designed for low income groups have been established all over the world. However, credit access for farmers in developing countries remains low. Digital financial services are rapidly expanding globally at the moment. They also bear great potential to address the credit needs of farmers in remote rural areas. Beyond mobile money services, digital credit is successively offered and also discussed in literature. Compared to conventional credit which is granted based on a thorough assessment of the loan applicant’s financial situation, digital credit is granted based on an automated analysis of the existing data of the loan applicant. Despite the potential of digital credit for serving the credit needs of rural farmers, empirical research on farmers’ willingness to pay for digital credit is non-existent. We employ a discrete choice experiment to compare farmers’ willingness to pay for digital and conventional credit. We apply loan attributes which reflect typical characteristics of both credit products. Our results indicate a higher willingness to pay for digital credit compared to conventional credit. Furthermore, we find that the proximity to withdraw borrowed money has a higher effect on farmers’ willingness to pay for digital credit compared to conventional credit. Furthermore, our results show that instalment repayment condition reduces farmers’ willingness to pay for digital credit whilst increasing their willingness to pay for conventional credit. Additionally, we find that longer loan duration has a higher effect on farmers’ willingness to pay for digital credit compared to conventional credit whereas higher additional credit cost has a lower effect on farmers’ willingness to pay for conventional credit compared to digital credit. Our results highlight the potential of digital credit for agricultural finance in rural areas of Madagascar if a certain level of innovation is applied in designing digital credit products.

## 1. Introduction

It is frequently reported that farmers in developing countries have a lower probability of credit access or are more credit constrained compared to non-agricultural firms [1–4]. Partly, this is due to the high transaction costs financial institutions have to incur in administering small loans to farmers in rural areas [5]. Digital finance (e.g. mobile money services, digital credit) presents an opportunity to increase credit access to farmers in developing countries, even in remote rural areas. This is possible because of the rapid spread of mobile phones in developing countries over the past decade [6] but also due to partnerships between financial institutions and mobile network operators (MNOs).

Previous studies on digital financial services in developing countries primarily focused on the impact of mobile money services on household consumption, income, and food security [7–10]. These studies generally showed a positive impact of mobile money services on household welfare. However, a branch of digital finance which lacks research concentration in developing countries is digital credit. According to Chen and Mazer [11], digital credit are loans that are “instant” (takes seconds or a maximum of 24 hours from application to loan decision), “automated” (uses digital data of borrowers to evaluate credit worthiness by credit scoring mechanisms), and “remote” (loan application, disbursement, and repayments happen with limited human interactions). “Instant, automated, and remote” differentiate digital credit from conventional credit based on the time to take decisions, risk management process, and sending information and payments. Digital data of borrowers include mobile phone airtime top-up and use, duration of calls, frequent usage of short message service, mobile money transactions, data top-ups, mobile wallet balance, previous loan status [12]. The limited number of existing studies on digital credit shows that digital credit can improve access to formal financial services to the previously unbanked and underserved population, even in rural areas [11–17]. For example, Francis et al. [13] looked at the current landscape of digital credit in Sub-Sahara Africa (SSA). The study identified low transaction costs, remote disbursement and repayment of loans, and the use of non-traditional data of potential borrowers’ to access credit worthiness as the key advantages of digital credit compared to conventional credit. These characteristics make digital credit a possible option for farmers in rural areas of developing countries to have credit access.

As the potential of digital credit to improve credit access to the unbanked population could be established, little is known about potential borrowers’ (e.g. farmers’) willingness to pay (WTP) for digital credit. Bridging this knowledge gap is necessary for financial service providers in developing countries to design digital credit products to serve potential borrowers accordingly. Up to now, there is no research paper on digital credit that investigates potential borrowers’ WTP for digital credit, specifically from the perspective of farmers in developing countries.

Therefore, the objective of this paper is to investigate farmers’ WTP for digital credit. In particular, this paper sheds light on whether farmers’ WTP for digital credit differs from their WTP for conventional credit. Furthermore, we investigate if loan duration, repayment condition, traveling distance, and additional credit cost (e.g. withdrawal fees) have a different effect on farmers’ WTP for digital credit compared to conventional credit. Moreover, it may be possible that farmers’ WTP for credit products could be influenced by their socio-economic characteristics, therefore, we account for the potential role of farmers’ socio-economic characteristics in choosing a credit product.

To our knowledge, this paper is the first to provide insight into WTP for digital credit. We focus our analysis on Madagascar because access to financial services is limited in rural areas of the country and for farmers in particular [18]. Furthermore, Madagascar is of a particular importance as only about 5.5% of the adult population has a bank account at a formal financial institution, and about 70% of the population (mainly farmers) live in rural areas [19]. This offers an interesting setting for the study. For this study, we use primary data collected from rural farmers in Madagascar. Our findings will help make adequate policy interventions to increase credit supply to farmers, but also help financial institutions for effective product design, consumer targeting, and to induce the adoption of digital credit products among farmers in rural Madagascar.

The rest of the paper is organized as follows: In Section 2, we provide a brief background of Madagascar and highlight the potential of digital credit for rural farmers in the country. This is followed by a description of the materials and methods used for the study in Section 3. Results and discussion are presented in Section 4, and Section 5 concludes the paper.

## 2. Madagascar context and the potential of digital credit for rural farmers

The study is set in Madagascar, one of the least developed countries in the world with about 75% of the population living below the poverty threshold of $ 1.90 a day in 2019 [20]. About 63% of the population are subsistence farmers who live in remote rural areas [21]. Infrastructural development such as road network and electricity are limited in rural areas [20,22]. As a result, formal financial institutions in the country are largely concentrated in urban areas with little presence in rural areas [18]. Consequently, a large number of people in rural areas, who are mainly farmers, are excluded from formal credit markets. A major reason often cited in literature for the limited presence of formal financial institutions in rural areas of developing countries is the higher administrative costs that financial institutions have to incur in administering small loans in rural areas [5,23]. Detailed client assessment for credit is demanding and time consuming for loan officers, especially in rural areas given that most farmers may not have sufficient records of their farm activities. In response to the information asymmetries, formal financial institutions normally demand collateral from loan applicants to reduce credit assessment costs and also to secure their investment [24,25]. However, for most farmers in rural Madagascar, collateral may not be available, thus, excluding them from formal credit markets.

Furthermore, conditions of conventional loans offered by formal financial institutions in the country have been identified as one of the main reasons for the perpetual lack of credit access to farmers in Madagascar [26]. Conventional loans have weekly/monthly repayment obligations which start soon after loan disbursement [27]. However, such repayment conditions are not suitable for agricultural production. Agricultural production is characterised by high seasonality and irregular cash flows [28]. This makes it very difficult for farmers to have credit access from formal financial institutions in times of need. Oftentimes, the only possibility for farmers in rural areas of the country to address their credit needs is to turn to non-formal sources (e.g. family and friends, informal money lenders), which sometimes, may not be available in times of need.

However, in recent years, digital credit has developed rapidly in some countries in SSA such as Madagascar, as an alternative for people who are normally excluded from the formal credit markets [12,15]. Unlike conventional credit which is granted based on a thorough assessment of the loan applicant’s financial situation, digital credit is granted based on an automated analysis of the digital data of the loan applicant. Normally, the supply of digital credit involves a partnership between an MNO and a bank [12,29]. The MNO manages the mobile money accounts of customers, agent networks (i.e. network for mobile money agents), and provides the digital data of customers to evaluate credit worthiness, whereas the bank provides the loans [15].

Digital credit has several advantages for farmers in rural Madagascar compared to conventional credit. First, the process from loan application to loan decision is “instant” [11], which makes it possible for farmers to have access to credit at the moment of need. Second, the automation of the credit evaluation process makes it possible for digital credit providers to expand credit to a large number of individuals without collateral, who may be excluded from formal credit markets [13], as the case of most farmers in rural areas of Madagascar. Third, loan applications, disbursements, and repayments can be managed remotely without making a trip to the bank [11]. Normally, a prerequisite for an MNO customer to apply for digital credit is that the customer must be registered for mobile money and should be an active user of the MNO for at least six months [12]. Once these two conditions are fulfilled, the customer can apply for a loan at any time through the customer’s mobile money account with the MNO. Following a loan application, the loan decision is instant and automated based on preset parameters [11,12].

Once a loan application is approved, disbursement is done remotely and the borrower can withdraw the loan at a nearby mobile money agent (a shop where one can change the digital money to physical money) subject to a withdrawal fee. The withdrawal fee is the amount of money that a digital credit borrower has to pay to a mobile money agent in order to change the digital money to physical money. Loans are usually smaller, shorter-term, and more expensive compared to conventional credit [12]. When it is time to repay the loan, borrowers have to make payments into their digital account (mobile money account) from a mobile money agent to repay the loan remotely.

The combination of these characteristics “instant, automated, and remote” underscores the ability of digital credit to address some, if not all, of the credit needs of farmers in rural areas of Madagascar. For example, the automation of the credit evaluation process reduces client’s assessment costs, and eliminates collateral requirements for credit evaluation. Additionally, the automation of the credit evaluation process makes it possible for digital credit providers to by-pass any potential information asymmetries that may arise from the loan applicant in credit evaluation. Furthermore, the “remote” nature of digital credit reduces infrastructural requirements and geographical distance for the provision and access to credit for farmers in rural areas. This makes digital credit particularly important for farmers in rural areas of Madagascar given the low penetration of formal financial institutions in rural areas of the country. Moreover, given that digital credit is “instant”, it is possible for farmers in rural areas to apply for credit at the moment of need without the need to spend considerable time and money to travel to the bank to apply for conventional credit. Digital credit can be beneficial for farmers in rural areas of Madagascar because previous studies have indicated the importance of credit access to farmers for farm investment, productivity and profitability [1,24,30]. Given the limited presence of formal financial institutions in rural areas of the country [18], digital credit can help farmers in rural areas of Madagascar to address the credit needs of their farm operations. For example, credit to purchase an improved variety of seeds for planting.

## 3. Materials and methods

### 3.1 Ethics statement

Our study uses data from a socio-economic survey of smallholder farmers in Madagascar. The institutional review board of the University of Goettingen only reviews clinical research; our study cannot be categorized as clinical research. We consulted the Head of the Research Department of the University of Goettingen, who confirmed that there is no institutional review board at the University of Goettingen that reviews socio-economic research.

We carried out a face-to-face interview with each of the farmers who participated in the survey with the help of highly trained enumerators, under the supervision of the researchers. Individual interviews were conducted in the local language of the respondents. Participation of farmers in the survey was voluntary. Before each interview, the enumerator explained the objectives of the research to the respondent. Further, the enumerators clarified that the data collected from the respondent during the interview would be confidential, and be used solely for scientific purposes. Based on this, the farmers’ were asked for their verbal consent to participate in the study. We opted for verbal consent instead of written consent from the participating farmers’ given that most of the participating farmers had only few years of formal education.

### 3.2 Data collection

The study uses primary data collected from smallholder farmers from the districts Ambatolampy, Ambohidratrimo, Arivoimamo, Betafo, Miarinarivo, and Tsiroanomandidy in central Madagascar between December 2019 and February 2020. We conducted interviews with smallholder farmers: some are clients and some are non-clients of a large commercial microfinance bank, Access Bank Madagascar (ABM). The inclusion of ABM clients (mainly farmers) in the study was advantageous because ABM is one of the largest microfinance banks in Madagascar and offers agricultural loans. Founded in 2007 with a strong mission towards financial inclusion for the poor, ABM seeks to expand its digital financial product range and allowed us to freely conduct our discrete choice experiment (DCE) within their business districts. However, at no moment was there any influence of ABM on our research, for example, in the selection of the sample for the study.

We applied a multi-stage sampling method to draw the sample for the study. At the first stage, we purposively selected six branches of ABM from six districts, one branch from each district. These branches were selected because they offer agricultural loans in predominantly agricultural communities. These six branches are located mainly in rural areas. For the selection of the non-ABM clients, we randomly selected two villages from each district. At the second stage, we randomly selected from each of the selected six ABM branches approximately 35 farmers who are clients of ABM for interviews. These clients were drawn from a complete list of clients on the agricultural loan portfolio of each branch. Similarly, for the selection of non-ABM clients for the study, in each of the two randomly selected villages at each district, 17 or 18 households were randomly selected from each village for interviews based on complete household lists. Consequently, a sample of 420 smallholder farmers, comprising of 210 ABM clients and 210 non-ABM clients drawn from 6 ABM branches and 12 villages were used for the study.

The sample for the study were predominantly smallholder farmers with a concentration on rice and vegetable (e.g. carrot, green beans, cucumber) production. Rice is the main staple food in Madagascar. These crops are grown mostly for household consumption. The questionnaire for the study begins with general questions about the respondent’s household. It then proceeds to the access to formal financial services (e.g. credit access), farm information, a DCE, and finally, it investigates farmers’ financial knowledge.

### 3.3 Conceptual framework

We employ a DCE to investigate farmers’ WTP for digital credit compared to their WTP for conventional credit. DCEs have been extensively used in the agricultural, development, health, and energy economics literature to elicit individuals’ preferences for goods and services [31–35]. Lancaster’s consumer theory and McFadden’s random utility theory serve as the basis for choice modelling [36,37]. The underlying principle is that consumers derive utility from the characteristics of a good instead of the good itself and consumers choose the good with the maximum utility among a set of alternatives.

Following Coffie et al. [31] and Hensher et al. [38], and consistent with random utility theory, we assume that a farmer *n* faces a choice among *J* credit products in choice situation *t*. The utility of farmer *n* from choosing alternative *i* in choice situation *t* can be partitioned into two components: an observed or modeled component, *V*(*X*_*nit*_, *β*_*n*_), and a residual unobserved and un-modeled component, Ԑ_*nit*_, such that:

$$U_{nit}=V_{nit}(X_{nit},\;\beta_{n})+{Ԑ}_{nit}$$ (1)

where *U*_*nit*_ is the utility a farmer *n* derives from choosing credit product *i* in choice situation *t*. *X*_*nit*_ is a vector of observed attributes of alternative *i* in choice situation *t*. *β*_*n*_ is a vector of parameters to be estimated which account for the farmer’s preferences for credit product attributes, and Ԑ_*nit*_ is the error term which is independently, identically distributed extreme value [39] of the expected utility that is not observed.

Following Hensher et al. [38], we assume that for a given choice set of credit products *J*, a farmer *n* in choice situation *t* will choose credit product *i* from *J* if and only if credit product *i* provides the maximum utility compared to any other alternative *j*. Therefore, the probability that a farmer *n* chooses credit product *i* from the possible credit products *J* in choice situation *t* is given by:

$$P_{nit}=Prob(U_{nit}>U_{njt},\forall i\neq j;\;i,j\in J)$$

$$={Prob(V}_{nit}+{Ԑ}_{nit}>V_{njt}+{Ԑ}_{njt},\;\forall i\neq j;\;i,j\in J)$$ (2)

### 3.4 Experimental design

DCEs underline the stated preference approach, which allows for conclusions to be drawn from previously unarticulated preferences about real choice decisions [40]. The attribute-based measurement of participants’ preferences is possible through a series of hypothetical decision-making situations [41]. In a DCE, participants are presented with a number of choice sets, each consisting of different alternatives, and are asked to select one of the given alternatives. Each presented alternative is characterized by pre-defined attributes and their associated levels. By systematically varying the attributes and their levels, the respective influence on the selection decision can be determined [40]. DCE is appropriate for our study because digital credit is new in Madagascar so there is no available data.

The DCE utilized in this study presented the following decision situation to the participating farmers: based on a labeled design, the farmers had to choose between a digital credit and conventional credit or could decide not to use either of these alternatives (opt-out). The opt-out alternative was included so that the choice for one of the proposed alternatives is voluntary. A forced choice could lead to inaccuracy and inconsistency with demand theory [42]. The details of the instructions for the farmers during the DCE are presented in supporting information S1 Text. The attributes for the experimental design and their levels were derived from the digital and conventional credit literature and a pilot study with 20 smallholder farmers in the study districts in October 2019. Following the pilot study, we identified five credit attributes that are important to farmers when choosing a credit product: loan duration, interest amount per month, repayment condition, traveling distance, and additional credit cost. Table 1 presents the alternatives, attributes and levels used in the experiment.

**Table 1:** Alternatives, attributes, and levels.

Alternatives	Attributes	Levels
*Digital credit*		
	Loan durarion	1 month; 3 months; 6 months
	Interest amount per month	MGA 12,000; MGA 16,000; MGA 20,000; MGA 24,000
	Repayment condition	1 = Instalment; 0 = At maturity
	Traveling distance	0.5 km; 1 km
	Additional credit cost (withdrawal fees)	MGA 2,000; MGA 6,000; MGA 10,000
*Conventional credit*		
	Loan duration	3 months; 6 months; 12 months
	Interest amount per month	MGA 8,000; MGA 12,000; MGA 16,000
	Repayment condition	1 = Instalment; 0 = At maturity
	Traveling distance	5 km; 10 km; 20 km
	Additional credit cost (transaction fees)	MGA 6,000; MGA 10,000; MGA 14,000

*Note*: MGA: Malagasy Ariary. Credit amount: MGA 200,000. 1 € = MGA 4,150.

The first attribute is loan duration, which is the time between when the borrower gets the money from the bank/MNO and pays it back to the lender. Digital credit is a short-term loan used by borrowers to manage small liquidity needs [13,29]. Most of the digital credit products in SSA have a duration of one month, even though a few digital credit products have a duration of up to six months [12,13,43]. For example, “M-Kajy”, in Madagascar, has a duration of one month whereas “M-Shwari”, and “KCB M-Pesa” (both in Kenya) has a duration of one month and a duration of up to six months, respectively. As a result, we selected one month, three months, and six months as the levels for the loan duration for digital credit. However, conventional credit products are inherently characterized by a longer loan duration compared to digital credit products. For example, the standardized loan duration for the Grameen Bank in Bangladesh is one year [44]. Additionally, conventional credit products are normally designed with different loan durations to serve the credit needs of borrowers [45]. For example, farmers may require different loan durations depending on the types of crop(s) they cultivate. Thus, for conventional credit, we selected three months, six months, and 12 months as the levels for loan duration. For farmers, the loan duration may be very important when choosing a credit product given that the duration should be long enough for farmers to make prudent production decisions on their farms.

The second attribute is interest amount per month, which is the cost of borrowing per month excluding the principal credit amount. Usually, this cost is expressed as interest rate per month. However, in this experiment, instead of interest rate per month, we refer to it as interest amount per month because we realized during the pilot study that the participating farmers found it difficult to understand and interpret percentage points. Hwang and Tellez [12] and Francis et al. [13] indicate that the interest rate of digital credit products is normally higher compared to conventional credit from a bank. For example, in Madagascar, “M-Kajy” and “MVola” has a monthly interest rate of 7% and 9%, respectively [43,46]. Following the pilot study, we selected four levels of interest amount per month for digital credit: MGA 12,000, MGA 16,000, MGA 20,000, and MGA 24,000. These levels represent 6%, 8%, 10%, and 12% of the credit amount of MGA 200,000, respectively. A credit amount of MGA 200,000 (€48) was selected for this experiment because in the study setting, the farmers considered this amount to be sufficient for their farm operations per production season, for example, credit to purchase improved seeds or pay workers at the start of the planting season. For conventional credit, the International Monetary Fund [47] reports an average interest rate of 55.4% per annum for conventional credit products for Madagascar. Given the limited presence of financial institutions in rural areas of the country, interest rate per annum on conventional credit products could possibly be higher in rural areas compared to the officially reported figures. Following the pilot study, we selected three levels of interest amount per month for conventional credit: MGA 8,000, MGA 12,000, and MGA 16,000. These levels represent 4%, 6%, and 8% of the credit amount, respectively.

In this study, it is important to note that interest amount per month is the main cost component of both credit products. This differs considerably from additional credit cost (e.g. withdrawal fees/loan processing fees) which is one-off cost to the borrower per loan application and especially applied for digital credit. For example, digital credit borrowers have to pay the interest amount per month through their mobile phone to an MNO whereas the withdrawal fees are paid to a mobile money agent for loan disbursement. Moreover, the additional credit cost is a very small amount of the total credit amount compared to the interest rate per month. In order to reflect the reality of farmers in our experiment when choosing a credit product, it was appropriate to separate interest amount per month from additional credit cost for both credit products. Thus combining both cost components in the experiment would have been inappropriate, and challenging for farmers to understand.

The third attribute is repayment condition, which indicates how a borrower has to repay the loan to the lender. For conventional credit, previous studies have suggested the provision of loans with flexible repayment conditions to farmers because of the seasonality of agricultural production [26,30,48]. However, for digital credit, little is known about borrowers preferences for repayment conditions in general and for farmers in particular. Hwang and Tellez [12] suggest that digital credit borrowers can repay their loans in instalments or at maturity. In this study, instalment repayment condition denotes that farmers can repay part of the principal credit amount and the interest rate per month at regular intervals (e.g. on monthly basis) until the farmer finishes paying the credit amount and the interest rate per month at the end of the loan duration. Alternatively, at maturity repayment condition denotes that farmers can defer repayment of the principal credit amount and the interest rate per month to the end of the loan duration. Thus, we use this binary attribute in our experiment to investigate farmers’ preference for repayment condition for both credit products.

The fourth attribute for our experiment is traveling distance, which indicates how long farmers have to travel in order to access the nearest formal financial institution or mobile money agent. Banks penetration in rural areas of Madagascar is particularly low [18], thus, people in rural areas have to travel for a considerable distance in order to access conventional credit in urban areas. The associated transaction costs can be substantial, which could prevent people in rural areas (mainly farmers) to access conventional credit [49]. In this study, the distance from a borrower’s house to the nearest formal financial institution is the traveling distance for conventional credit. Following the pilot study, we set the traveling distance for conventional credit to three levels: 5 kilometers, 10 kilometers, and 20 kilometers. Even though digital credit is “remote”, bridging geographical distance, it should be noted that once a loan application is approved, before the borrower can withdraw the loan, they typically have to travel for a short distance (e.g. 500 meters) from their house to a nearby mobile money agent. This distance is the traveling distance for digital credit borrowers. Based on the pilot study, we selected two levels for traveling distance for digital credit: 0.5 kilometers and 1 kilometer.

The fifth attribute of our experiment relates to the additional credit cost that has to be incurred by borrowers apart from the interest amount per month. For example, digital credit borrowers have to pay a withdrawal fee to a mobile money agent in order to withdraw a digital loan. Mobile money agents offer cash-in/cash-out services [50], which are essential for the deployment of digital credit. Even though there is little research evidence to suggest the withdrawal fees that borrowers have to pay in order to disburse their digital loans, we selected three levels of additional credit cost for digital credit following the pilot study: MGA 2,000, MGA 6,000, and MGA 10,000. These levels represent 1%, 3%, and 5% of the credit amount, respectively. Similarly, conventional credit borrowers have to pay additional credit costs apart from the interest amount per month. For example, they have to pay loan processing fees [51], and withdrawal fees if they withdraw their loans through an automated teller machine [52]. Following the pilot study, we selected three levels of additional credit cost for conventional credit: MGA 6,000, MGA 10,000, and MGA 14,000. These levels represent 3%, 5%, and 7% of the credit amount, respectively. It is important to note that in order to improve farmers’ understanding of the experiment; instead of percentage points, we used absolute figures for the additional credit cost for both credit products. This was necessary because we observed during the pilot study that the farmers found it difficult to understand percentage points.

In this study, a labeled DCE is preferred because it is the best method to directly analyse the trade-off between digital and conventional credit. This is because in this study, a labeled design allows focusing on the main effects of the two credit products as each credit product conveys information itself for the farmer based on their experience and knowledge [53,54]. Furthermore, labeled designs offer less abstract choices to respondents and this may add to the validity of the results [54]. Additionally, labeled choice experiments allow using alternative specific attributes or levels [38,55]. In our case, this was necessary as especially the levels for the attributes “loan duration”, “interest rate”, “traveling distance” and “additional credit cost” are alternative specific. For example, it was not realistic to combine the alternative digital credit with a traveling distance of 20 kilometers. In particular, this approach made it possible to take different cost levels into account which is very meaningful when comparing digital and conventional credit.

In this study, the trade-off for farmers to choose either credit product is based on the timely availability of the credit product (instant availability vs. waiting for several days), credit evaluation criteria (automation of credit decision vs. loan officer’s judgement), mode of sending information and payments (remote vs. in person), and the credit characteristics (e.g. interest amount per month, loan duration, traveling distance). For example, digital credit borrowers can manage loan applications, disbursements, and repayments remotely without making a trip to a bank. However, such convenience generally comes with a loan product with a higher interest rate per month and a shorter loan duration compared to conventional credit products [12]. Similarly, conventional credit borrowers may pay lower interest rate per month and have loans with longer duration compared to digital credit borrowers, however, they have to spend considerable time and money to travel several kilometers from their place of residence to a nearby town to access conventional credit. Additionally, the credit evaluation criterion for conventional credit is based on a detailed assessment of the loan applicant’s business data by a loan officer, which may often not be available for rural farmers in the study districts whereas that of digital credit is automated based on the digital data of the loan applicant, which is generally available for all mobile phone users.

Important to highlight in this experiment is the selection of different figures and levels for the same attribute for both credit products (e.g. interest amount per month, traveling distance). This is prominent in the case of the attribute “traveling distance”. However, this is necessary to reflect the credit characteristics of both credit products in the study setting. For example, in the study setting, the traveling distance for digital credit is a walking distance whereas that of conventional credit is a vehicular distance, hence, attracting different figures and attribute levels in our experiment.

It is important to indicate that even though digital credit differs from conventional credit [11], however, in the study setting, a direct comparison of farmers’ WTP for both credit products is plausible for two reasons. First, formal financial services in Madagascar are largely concentrated in urban areas with very little presence in rural areas. As a result, farmers in rural areas have to spend considerable time and money to travel to the nearest town/community with a bank/microfinance institution (MFI) in order to apply for conventional credit if needed. This makes digital credit particularly important for rural farmers in the study setting. Second, this study is designed to serve the credit needs of smallholder farmers in rural areas of Madagascar who require a small credit amount per production season for farm operations.

Building a full-factorial design with the number of alternatives, attributes and levels presented in Table 1 results in [(4·3·2·2·3)Digital credit · (3·3·2·3·3)Conventional credit =] 23,328 possible decision situations or choice sets. However, for the sake of practicability, this design was determined to be too extensive and therefore, the number of choice sets was reduced. To minimize the simultaneous and unavoidable loss of information when reducing the full factorial design, a so-called “efficient design” was applied. As a result, a D-efficient Bayesian design [56,57] with 12 choice sets blocked into two groups of six each were found to be appropriate for the study following the pilot study. Thus, each of the participating farmers in the main survey answered six choice sets. The six choice sets in each block (blocks 1 and 2) as presented to the farmers during the main survey are presented in supporting information S1 Table.

### 3.5 Estimation procedure

In order to investigate farmers’ WTP for digital credit compared to their WTP for conventional credit, first, we determine the factors that influence farmers’ preferences for either digital or conventional credit. For this purpose, we apply the mixed logit model [58]. The mixed logit model relaxes the restrictive independence of irrelevant alternatives assumption of the conditional logit model. McFadden and Train [59] suggested that the mixed logit is a very flexible model that can estimate any random utility model. It addresses the shortcomings of the standard logit model by allowing for correlation of unobserved factors over time, unrestricted substitution patterns, and taste parameters to vary across individuals [39]. From Eq (1), we model the utility of a farmer *n* from choosing credit product *i* among *J* credit products in choice situation *t* as:

$$U_{nit}={ASC}_{i}+{\beta^{\prime }}_{n}X_{nit}+{Ԑ}_{nit}$$ (3)

where *U*_*nit*_ is the utility a farmer *n* associates with choosing credit product *i* in choice situation *t*. *ASC*_*i*_ is the alternative specific constant of alternative *i* which accounts for the average effect of all the factors that are not included in the model on utility [39]. *X* is a vector of alternative specific credit product attributes, which include loan duration, interest amount per month, repayment condition, traveling distance, and additional credit cost; *β*_*n*_ are the associated parameters to be estimated for each of the credit product attributes; and Ԑ_*nit*_ is the error term which is distributed independent and identically distributed extreme.

Even though it is established that the mixed logit model accounts for preference heterogeneity among individuals [38,39], Boxall and Adamowicz [60] suggest that the mixed logit may be constrained when explaining the sources of heterogeneity. They suggest that in many instances, the sources of heterogeneity relate to the socio-economic characteristics of the individual decision maker. Therefore, to account for the potential role of the socio-economic characteristics of a farmer *n* in choosing a credit product *i* in choice situation *t*, Eq (3) is slightly modified to estimate a mixed logit model of the form:

$$U_{nit}={ASC}_{i}+{\beta \prime }_{n}X_{nit}+\mu \prime ({ASC}_{i}\;x\;S_{n})+{Ԑ}_{nit}$$ (4)

where (*ASC*_*i*_ x *S*_*n*_) is a vector of variables accounting for the interactions of smallholder farmers’ socio-economic characteristics *S*_*n*_ (e.g. age, risk attitude) and the *ASC*_*i*_ associated with the choice of credit product made by a farmer *n*; μ are the associated coefficients to be determined.

We use Stata 15 to apply the simulated maximum likelihood estimator with 1,000 Halton draws to estimate the mixed logit model [58]. Following Hensher and Green [61], the main price attribute (i.e. interest amount per month) in the experiment is estimated as a non-random parameter; otherwise it could result in unrealistic WTP estimates. Further, in the estimation of the mixed logit model, the attributes: interest amount per month, loan duration and additional credit cost for each credit product are modeled as continuous variables based on the attributes levels. A similar argument could be presented for the attribute “traveling distance” for conventional credit. However, given the levels of the attribute “traveling distance” for digital credit compared to conventional credit in our experiment, we modeled the attribute “traveling distance” for digital credit as a linear variable in order to avoid any potential collinearity between the traveling distance for digital credit and the constant for digital credit. Furthermore, the attribute “repayment condition” for both credit products is modeled as effects-coded variables. Similarly, all farmers’ socio-economic characteristics except age, years of education, and their risk attitude are modeled as effects-coded variables. Following Hensher et al. [38], we preferred effects-coding to dummy coding in the estimation of the mixed logit models in order to avoid confounding of the base attribute level with the grand mean of the utility function.

In order to estimate farmers’ WTP for the different attributes of each credit product, we follow Train and Weeks [62] to re-specify Eq (3) to indicate the difference between the main price attribute (interest amount per month), *P*_*nit*_, and the other attributes (loan duration, repayment condition, traveling distance, additional credit cost), *X*_*nit*_:

$$U_{nit}={{ASC}_{i}-\alpha }_{n}P_{nit}+{\beta \prime }_{n}X_{nit}+{Ԑ}_{nit}$$ (5)

Accordingly, we apply the Krinsky and Robb method using the Stata module *wtp* [58] with 10,000 replications to estimate farmers’ WTP for credit products attributes. From Eq (5), the ratio of an attribute’s coefficient (*β*_*n*_) to the price coefficient (*α*_*n*_) is the WTP for that attribute:

$${WTP}_{n}=\frac{\beta_{n}}{\alpha_{n}}$$ (6)

Consequently, we follow Hensher et al. [38] to apply the Wald test to verify if the difference between corresponding WTP estimates for digital and conventional credit attributes is statistically significantly different from zero.

## 4. Results and discussion

### 4.1 Descriptive statistics

Table 2 shows the summary statistics describing the socio-economic characteristics of the sampled farmers. The mean age of the farmers is about 39 years. The sampled farmers have to travel on average 10 kilometers to the nearest formal financial institution to access financial services (e.g. credit), a condition which highlights the low penetration of banks/MFIs in rural areas of Madagascar. Further, it is observed that during the past 12 months, only 34% of the farmers had their application for credit from a formal financial institution approved. However, a higher number (51%) is reported if we consider credit to farmers from both formal and non-formal sources. We also notice from Table 2 that majority of the farmers have access to a mobile phone (88%), a device necessary for the use of digital credit. Mobile phone subscription rate in Madagascar is about 27% [63], however, we report a higher mobile phone subscription rate in our sample given that we focused on mobile phone availability in the farmers’ households. In the study context, both simple phone and smartphone are suitable for digital credit operation. This makes the use of digital credit feasible for farmers in the study districts. Further, Table 2 shows that the farmers have to travel on average one kilometer in order to access a mobile money agent. Additionally, it emerged that the sampled farmers have a mean monthly income of about MGA 414,008 (approximately €100) and five persons per household.

**Table 2:** Summary statistics of respondents.

Variable	Unit	Mean	SD
Age	Years	39.395	13.141
Credit access (Yes)	1/0	0.343	-
Distance to the nearest formal financial institution	Kilometers	9.558	12.588
Distance to the nearest mobile money agent	Kilometers	0.935	0.468
Education	Years	9.888	4.260
Farming experience	Years	14.865	12.334
Financial knowledge ^a)^	Number	4.186	1.285
Gender (Male)	1/0	0.540	-
Household size	Number	4.574	1.691
Land size (Owned land)	Acres	2.945	2.392
Marital status (Married)	1/0	0.867	-
Mobile phone access (Yes)	1/0	0.876	-
Monthly income	MGA	414,008	223,100
Received credit from any source (Yes)	1/0	0.507	-
Remittances (Yes)	1/0	0.374	-
Risk attitude ^b)^	Number	6.790	1.955
Number of participants		420	

*Note*: MGA: Malagasy Ariary. 1 € = MGA 4,150. Mean values for dummy variables (1/0) indicate ratios.

^a)^ Measured on a scale from 1 (very low financial knowledge) to 7 (very high financial knowledge) (cf. Lusardi and Tufano, [64]).

^b)^ Measured on a scale from 1 (risk averse) to 11 (risk seeking) (cf. Dohmen et al., [65]).

### 4.2 Farmers’ preferences and WTP for credit attributes

Table 3 presents the estimation results for the determinants of farmers’ preferences for credit products, accounting for their socio-economic characteristics and the calculated WTP for credit product attributes. We also estimated a model without socio-economic characteristics of the farmers. However, comparing the log-likelihood and AIC values of both models, it could be argued that the inclusion of the socio-economic characteristics of farmers through the interactions with the alternative specific constant of each credit product increases model performance. Therefore, for the purpose of our study, we interpret and discuss the results of the model with socio-economic characteristics. The model without farmers’ socio-economic characteristics is presented in Table A1 of supporting information S2 Table. Additionally, due to potential collinearity between the attribute “traveling distance” for digital credit and the constant for digital credit, we also estimated a model without the attribute “traveling distance” for both credit products to check the robustness of our results. We report the results in Table A2 of supporting information S3 Table. The results are largely consistent with the results of Table 3. Furthermore, Table 4 presents the results of the Wald test indicating the difference between corresponding mean WTP estimates for digital and conventional credit attributes. Based on these results, we focus on the mean WTP estimates to evaluate whether farmers’ WTP for digital credit differs from their WTP for conventional credit. Similarly, we evaluate whether credit product attributes (loan duration, repayment condition, traveling distance, and additional credit cost) have a different effect on farmers’ WTP for digital credit compared to conventional credit.

**Table 3:** Determinants of farmers’ preference for credit products estimated by the use of a mixed logit model.

Variable	Mean coefficient	SD coefficient	Mean WTP	Minimum WTP	Maximum WTP
	(Standard error)	(Standard error)	in MAG	in MGA	in MGA
***Digital credit***					
Constant	3.230***	0.791***	25,659	9,781	42,196
	(1.066)	(0.267)			
Loan duration	0.129***	-	1,028***	470	1,565
	(0.037)				
Interest amount per month	-0.013***	-	-	-	-
	(0.001)				
Repayment condition (Instalment = 1)^a)^	-0.229***	-	-1,815***	-3,213	-681
	(0.070)				
Traveling distance	-0.728***	-	-5,783***	-10,061	-1,868
	(0.255)				
Additional credit cost (Withdrawal fees)	-0.013***	-0.012***	-102***	-145	-66
	(0.002)	(0.004)			
***Conventional credit***					
Constant	3.521***	-	18,677	8,053	29,696
	(1.068)				
Loan duration	0.012	0.120***	63	-217	361
	(0.027)	(0.026)			
Interest amount per month	-0.019***	-	-	-	-
	(0.002)				
Repayment condition (Instalment = 1) ^a)^	0.323***	0.767***	1,711	879	2,680
	(0.078)	(0.105)			
Traveling distance	-0.063***	-	-333	-486	-209
	(0.012)				
Additional credit cost (Transaction fees)	-0.001	-	-3	-24	17
	(0.002)				
***Interaction variables***					
***Digital credit***					
Constant x Age	-0.027*	-			
	(0.015)				
Constant x Education	0.118**	-			
	(0.048)				
Constant x Mobile phone access ^a)^	0.727***	-			
	(0.251)				
Constant x Received credit ^a)^	-0.520**	-			
	(0.233)				
Constant x Risk attitude	0.538***	-			
	(0.113)				
***Conventional credit***					
Constant x Age	0.003	-			
	(0.015)				
Constant x Education	0.082*	-			
	(0.048)				
Constant x Mobile phone access ^a)^	0.648***	-			
	(0.250)				
Constant x Received credit ^a)^	-0.638***	-0.861***			
	(0.234)	(0.238)			
Constant x Risk attitude	0.475***	-			
	(0.113)				
Participants/Observations	420/7,560				
*Goodness of fit measures*					
AIC	3,092.203				
BIC	3,279.330				
Log likelihood	-1,519.102				
LR-Statistic (*χ*^*2*^) (5 d.f.)	248.090				
Prob > chi2	0.000				

Note

***, **, and * indicates statistical significance at the 1%, 5% and 10% levels, respectively. For mean WTP estimates, significance level is for the difference in farmers’ mean WTP between digital credit and conventional credit attributes. We report WTP estimates of non-significant attributes for the sake of comparison. All WTP values are in MGA. MGA: Malagasy Ariary. 1 € = MGA 4,150. SD indicates standard deviation. Only SD coefficients with statistical significance at the 1%, 5% and 10% levels are shown. The sign of the estimated standard deviations is irrelevant: Interpret them as being positive. ^a)^ Indicates effects-coded variable. Halton draws = 1,000. Krinsky replications = 10,000.

**Table 4:** Wald test proving difference in coefficients for both credit products.

Test	Wald chi-square	Prob > chi2
	statistic	
Digital = Conventional (constants)	0.15	0.702
Digital loan duration = Conventional loan duration	14.67***	0.000
Digital instalment repayment = Conventional instalment repayment	33.06***	0.000
Digital traveling distance = Conventional traveling distance	6.85***	0.009
Digital additional credit cost = Conventional additional credit cost	22.07***	0.000

Note

***, **, and * indicates statistical significance at the 1%, 5% and 10% levels, respectively.

#### 4.2.1 Farmers’ overall WTP for digital and conventional credit

We observe from Table 3 that the constants of both credit products are positive and statistically significant, suggesting that smallholder farmers prefer to choose either digital credit or conventional credit relative to no credit (opt-out). Further, we observe that relative to no credit (opt-out), farmers’ mean WTP for digital credit is MGA 25,659 (€6.13) per month compared to MGA 18,677 (€4.50) for conventional credit. Relating both WTP values to the principal credit amount (MGA 200,000), the results suggest that farmers’ are on average willing to pay an amount equivalent to 12.8% per month for digital credit compared to 9.3% per month for conventional credit. We associate this finding to the characteristics of digital credit (“instant, automated and remote”) compared to conventional credit, and the limited access to formal financial institutions in rural areas of Madagascar. It has been established that about 70% of formal financial institutions in Madagascar are located in urban areas [18] even though majority of the population live in rural areas [21]. Thus, most people in rural areas do not have access to formal financial institutions, and hence, are excluded from formal credit markets. This makes digital credit particularly important for people in rural areas of the country. Therefore, it is not surprising that farmers in the study districts are willing to pay more (e.g. interest payment) for digital credit per month compared to conventional credit although we notice from Table 4 that this difference is not statistically significantly different from zero (Wald chi-square statistic of 0.15). The finding on farmers’ WTP for digital credit is relatively higher than the interest rate per month for digital credit products offered in Madagascar. For example, “MVola”, charges a fixed interest rate of 9% per month whereas “M-Kajy” charges an interest rate of 7% per month [43,46]. However, our result is reasonable given the limited availability of formal financial institutions in rural areas of the country [18], but also due to the fact that some digital credit products in SSA are more expensive. For example, Equitel Eazzy Loan (in Kenya) charges an interest rate of 14% per month [13]. Initially, there is the inclination to argue that our finding on farmers’ WTP for conventional credit per month is high. However, our finding is plausible given that the lending rate for conventional credit in Madagascar can be as high as 55.4% per annum [47]. Despite the potential of digital credit to expand credit to farmers in rural areas of the study districts, it is important to mention that the higher interest rate per month for digital credit may render digital credit non-attractive for some farmers in the study area given the higher poverty rate in the country [20]. From Tables 3 and 4, we can conclude that farmers’ WTP for digital credit is higher than their WTP for conventional credit even though this difference is not statistically significantly different from zero.

#### 4.2.2 Loan duration (short vs. long duration)

The findings in Table 3 show that loan duration has a positive and statistically significant effect on farmers’ preference for digital credit. For conventional credit, loan duration is not statistically significant. However, the statistical significance of the standard deviation coefficient of loan duration for conventional credit suggests that some farmers in the study area consider loan duration as an important attribute when choosing conventional credit. These findings suggest that the loan duration for digital credit may be too short to support farmers’ production season compared to the loan duration for conventional credit. This is plausible because most of the farmers in the study area produce rice and vegetables which require between three and six months from planting to harvesting. Thus, increasing the loan duration of digital credit products to accommodate farmers’ production season will increase farmers’ preference for digital credit. Our finding on farmers’ preference for digital credit with longer duration is consistent with Kaffenberger et al. [15] who suggest that increasing the loan duration of digital credit products could help borrowers to use digital credit for productive purposes.

Further, it emerged from Table 3 that increasing loan duration by one month increases farmers’ WTP for digital credit by MGA 1,028 (€0.25) compared to MGA 63 (€0.02) for conventional credit. We attribute this finding to the characteristics of digital credit compared to conventional credit, and its ability to increase credit access to farmers in rural areas of the study districts. Further, the loan duration for most digital credit products in Madagascar is one month [43,46], which may not be sufficient for farmers in the study area to make prudent production decisions, thus, causing farmers to pay substantially more for an increase in the loan duration for digital credit compared to conventional credit. We confirm this finding by the results of the Wald test in Table 4 which show that the difference between farmers’ mean WTP for one month increase in loan duration for digital compared to conventional credit is statistically significantly different from zero at 1% significance level (Wald chi-square statistic of 14.67). Thus, from Tables 3 and 4, we can state that loan duration has a statistically significantly higher effect on farmers’ WTP for digital credit compared to conventional credit.

#### 4.2.3 Repayment condition (instalment vs. at maturity repayment)

We notice from Table 3 that instalment repayment has a negative and statistically significant effect on farmers’ preference for digital credit, suggesting that farmers prefer at maturity repayment to instalment repayment for digital credit, a finding which supports the seasonality of agricultural income. This finding is in line with Kaffenberger et al. [15] who suggest that digital credit needs to offer repayment conditions adapted to the irregular cash flows of farmers and casual workers to increase uptake among this segment of the population. However, for conventional credit, instalment repayment condition has a positive and statistically significant effect on farmers’ preference, a finding contradictory to the support for the provision of flexible loans to farmers in the literature [26,30,48]. However, instalment repayment could be plausible for farmers if they engage in farm activities with continuous returns (e.g. dairy farming).

Further, we observe from Table 3 that instalment repayment on average decreases farmers’ WTP for digital credit by MGA 1,815 (€0.44) whereas it increases farmers’ WTP for conventional credit by MGA 1,711 (€0.41). This suggests that offering at maturity repayment condition for digital credit will increase farmers’ WTP for digital credit. From Table 4, the results of the Wald test suggest that the difference between farmers’ mean WTP for instalment repayment for digital and conventional credit is statistically significant at 1% significance level (Wald chi-square statistic of 33.06). Thus, from Tables 3 and 4, we can conclude that instalment repayment condition has a statistically significantly lower effect on farmers’ WTP for digital credit compared to conventional credit.

#### 4.2.4 Traveling distance (short vs. long distance)

Our findings from Table 3 indicate that traveling distance has a negative and statistically significant effect on farmers’ preference for digital and conventional credit. These findings suggest that the longer the traveling distance the lower the preference for digital/conventional credit compared to no credit (opt-out). For both credit products these results are plausible: Inasmuch as farmers want to use digital credit, they do not want to travel for long distance from their houses to the nearest mobile money agent. Thus, increasing the number of mobile money agents in farmers’ neighborhood to reduce the traveling distance to mobile money agents could raise the interest of farmers in digital credit. For conventional credit, longer traveling distance presupposes that farmers in the study districts have to spend considerable time and money to travel to the nearest formal financial institution for conventional credit. In fact, it is reported that on average, people in rural areas of Madagascar have to travel for about 70 minutes in order to access the nearest financial institution [66]. However, the remoteness of digital credit makes it possible for farmers in the study districts to access credit without the need to spend such considerable time to travel. Our finding is in line with Karlan et al. [49] who suggest that transaction costs may limit access to formal financial services by people in rural areas.

When looking at farmers’ WTP for traveling distance for digital compared to conventional credit, we observe that farmers’ WTP for traveling distance for digital credit is -5,783 MGA (-€1.40) for a distance of 1 km. As this variable is linear, the value for a distance of 0.5 km is -2,892 MGA (-€0.70) (0.5·WTP_distance 1km_). This means that farmers prefer shorter traveling distance for digital credit. For conventional credit, an increase in the traveling distance by 1 km results in a decrease of farmers’ WTP by 333 MGA (€0.08). For example, if the traveling distance for conventional credit is 10 km, farmers’ WTP decrease by 3,330 MGA (€0.80) compared to 0 km. If we compare farmers’ WTP for digital to conventional credit, it can be stated that the traveling distance for digital credit has a higher effect on the farmers’ choice for a credit product until the decrease in farmers’ WTP for conventional credit exceeds the WTP_digital_ of 5,783MGA (€1.40). To calculate this point, we divide 5,783MGA (€1.40) by 333 MGA (€0.08), which yields a value of 17.4. This means that if the traveling distance for conventional credit is shorter than 17.4 km, farmers have a higher WTP for conventional credit compared to digital credit. However, if the traveling distance for conventional credit is longer than 17.4 km, farmers have a higher WTP for digital credit compared to conventional credit. This is plausible considering the traveling time and the accompanying transportation cost that a farmer has to incur in order to travel to the nearest formal financial institution for conventional credit if the traveling distance exceeds 17.4 km. Further, we observe from Table 4 that the difference between farmers’ mean WTP for traveling distance for digital and conventional credit is statistically significant at 1% significance level (Wald chi-square statistic of 6.85). The findings from Tables 3 and 4 suggest that traveling distance has a statistically significantly higher effect on farmers’ WTP for digital credit compared to conventional credit.

#### 4.2.5 Additional credit cost (withdrawal fees vs. loan processing fees)

We observe from Table 3 that additional credit cost has a negative and statistically significant effect on farmers’ preference for digital credit. This suggests that farmers are sensitive when fees (e.g. withdrawal fees) in addition to the interest rate are charged, a finding which should be of interest to financial service providers if digital credit is to be successful in rural areas. However, for conventional credit, the results indicate that additional credit cost has no statistical significant effect on farmers’ decision to choose conventional credit. Further, we observe that increasing the additional credit cost by MGA 1 (€0.00) decreases farmers’ WTP for digital credit by MGA 102 (€0.02) compared to MGA 3 (€0.00) for conventional credit. We associate this finding to the fact that farmers have to pay the withdrawal fees to a mobile money agent every time they have to change the digital money to physical money compared to loan processing fees for conventional credit which is a very small amount of the total credit amount, and paid per loan application.

The additional credit cost (e.g. withdrawal fees) can be important for use of digital credit in rural areas as a limited number of mobile money agents could incentivize mobile money agents to charge exorbitant fees from digital credit borrowers. Furthermore, it emerged from Table 4 that the difference between farmers’ mean WTP for MGA 1 (€0.00) increase in additional credit cost for digital and conventional credit is statistically significant at 1% significance level (Wald chi-square statistic of 22.07). Thus, from Tables 3 and 4, we can conclude that additional credit cost has a statistically significantly higher effect on farmers’ WTP for digital credit compared to conventional credit.

### 4.3 Effect of farmers’ socio-economic characteristics on their preference for a credit product

We observe from Table 3 that a farmer’s age has a negative and statistically significant effect on their preference for digital credit. The mean age of the sampled farmers is about 39 years, and they are less likely to be technologically inclined given the limited access to electricity in rural areas of the country [20]. This finding is in line with Cook and McKay [29] who suggested that the users of digital credit were more likely to be young (under the age of 35). Further, we notice that farmers’ years of education has a positive and statistically significant effect on their preference for digital credit. This is expected given that digital credit inherently requires some level of reading and numerical literacy skills to operate. Adult (15 years and older) literacy rate in Madagascar is about 75% and 68% for males and females, respectively [67]. This makes it possible for digital credit to thrive in Madagascar, even for farmers in rural areas. Our finding is consistent with Totolo [16] who suggested that the users of digital credit were more likely to be educated. We further observe from the model that farmers’ risk attitude has a positive and statistically significant effect on farmers’ preference for digital and conventional credit. This suggests that less risk averse farmers are more likely to choose either digital or conventional credit relative to no credit than more risk averse farmers. This is expected in the study setting given the limited presence of formal financial institutions in rural areas of the country [18]. Further, it emerged from the model that mobile phone access has a positive and statistically significant effect on farmers’ preference for digital and conventional credit. This is expected for digital credit as mobile phone access is one prerequisite for the use of digital credit. The lower subscription rate of mobile phones in Madagascar [63] highlights the need for strategies to increase mobile phone subscription in the country if digital credit is to be used by majority of people in the country, and for those in remote rural areas in particular. Further, we notice that farmers who received credit from any source (including from non-formal sources) over the past 12 months are less likely to use either digital or conventional credit, suggesting that a farmer who received credit from any source has no reason to seek for alternatives, particularly, in light of the excessively high interest rate per month for digital credit, and the traveling distance associated with conventional credit in the study setting.

## 5. Conclusion

People in rural areas of developing countries still lack access to credit, particularly, farmers. Digital credit is a recent innovation that has the potential to improve the situation. However, there is no literature which deals with farmers’ WTP for digital credit compared to conventional credit in developing countries. We employ a DCE to investigate farmers’ WTP for digital and conventional credit in rural Madagascar. We thereby consider different credit product attributes (loan duration, interest amount per month, repayment condition, traveling distance, and additional credit cost) which are important to farmers in the study area when choosing a credit product.

The results show that for a given credit amount, on average, farmers’ WTP for digital credit is higher compared to conventional credit. Furthermore, we find that the proximity to withdraw the borrowed money has a higher effect on farmers’ WTP for digital credit compared to conventional credit. Our results show that instalment repayment condition reduces farmers’ WTP for digital credit whereas it increases their WTP for conventional credit. Additionally, longer loan duration and higher additional credit cost have a higher effect on farmers’ WTP for digital credit compared to conventional credit.

Our results show the potential of digital credit for addressing the credit needs of farmers in rural areas of Madagascar if a certain level of innovation, for example, in repayment condition is met. With our findings, we can encourage financial service providers (e.g. MFIs, MNOs) in Madagascar to design digital credit products with loan duration sufficient enough to accommodate the production season of farmers. Moreover, offering at maturity repayment condition and charging withdrawal fees proportional to the disbursement amount regardless of the location of the mobile money agent should be considered. From our findings, we think that for digital credit to be successful among farmers in rural areas of the study districts, and Madagascar in general, it needs more than the three characteristics “instant”, “automated”, and “remote” [11]. Offering credit products which are not well adapted to farmers’ production needs will not be sufficient. Additionally, the sensitivity of farmers towards the additional credit cost of digital credit shows that the applied fee-practice of mobile money transfers might not be transferable to digital credit [63]: a transparent all costs including interest rate seems to be preferred, and hence, should be achieved by digital credit suppliers. Independent from who is offering digital credit, our results show that increasing the number of mobile money agents in farmers’ neighborhood could be important for the success of digital credit among farmers in Madagascar. From the perspective of farmers, our study suggest that improving the education of farmers (e.g. through adult education programs) could help rural farmers take advantage of financial service products such as digital credit when they are available. Additionally, strategies geared towards improving mobile phone accessibility and mobile network availability, particularly, in rural areas should be championed to facilitate the use of digital credit among farmers in Madagascar.

From a policy viewpoint, our results suggest that applying responsible finance standards like transparent product pricing without hidden costs can contribute to leverage the potential of digital credit for agricultural finance. Hence, the application of responsible finance standards should be advocated for formal financial institutions and digital financial service providers like MNOs. Finally, policy interventions could be geared towards educating farmers about new financial service products (e.g. digital credit) which could help farmers to address their small credit needs given the limited penetration of formal financial institutions in rural areas of Madagascar.

Following the ongoing discussions about the reliability and validity of DCEs [68,69], we have used two measures to make sure that the findings from our DCE are reliable and valid. The first measure is theoretical validity, which examines whether participants’ choices in a DCE follows the standard assumptions of rational choice theory [69,70]. To this effect, the findings from our DCE show that the farmers were rational in their decision making. We confirm this assertion with the negative coefficients of the attributes “interest amount per month”, “traveling distance”, and “additional credit cost” for both credit products in Table 3. The second measure used to check the validity of the findings of our DCE is criterion validity, which measures the degree to which preferences elicited from a DCE are related to a “criterion” which is deemed to be “true”, or largely closer to the construct under investigation [69,71]. Following this measure, we observe a higher WTP for digital credit per month in our DCE compared to the interest rate per month for digital credit products in Madagascar. Nonetheless, our results are plausible given the limited presence of formal financial services in rural areas of Madagascar.

However, as with other field experiments, this study has some limitations. First, the use of effects-coding for the variable “traveling distance” for digital credit in the choice design may have some effect on our estimation outcome. As such, future studies should consider using linear coding in the choice design. Second, the use of absolute figures instead of percentage points for the attributes “interest amount per month” and “additional credit cost” for both credit products may be crucial for the findings of the study. As a result, future studies for farmers in developing countries should consider the cognitive ability of farmers when designing DCEs. The use of control questions might be helpful in this regard.

Future studies on digital credit could focus on farmers’ preferences for digital credit with longer loan duration. Finally, this study is focusing on Madagascar; therefore, future studies on farmers’ WTP for digital credit could focus on other countries in SSA, as the conditions in Madagascar may not be applicable in the context of other countries.

## Supporting information

S1 Table Choice sets (Blocks 1 and 2). (DOCX)

S2 Table Determinants of farmers’ preference for credit products estimated by the use of a mixed logit model without accounting for socio-economic characteristics of farmers. (DOCX)

S3 Table Determinants of farmers’ preference for credit products estimated by the use of a mixed logit model without the attribute “traveling distance” for both credit products. (DOCX)

S1 Text Instructions for the respondent. (DOCX)

S1 File (DTA)
