# Supplementary material for: Farmers’ willingness to pay for digital and conventional credit: Insight from a discrete choice experiment in Madagascar
Source: PLoS One. 2021 Nov 12;16(11):e0257909. doi: 10.1371/journal.pone.0257909 (PMC8589200; doi:10.1371/journal.pone.0257909)
Supplement: S1 Table — (DOCX) [file pone.0257909.s001.docx]

**Choice sets (Blocks 1 and 2)**

**Block 1**

| Choice set 2 | | | |
| --- | --- | --- | --- |
| Attribute | Digital credit | Conventional credit | Opt-out |
| Loan duration | 1 month | 6 months | I prefer no |
| Interest amount per month | MGA 24,000 | MGA 16,000 | credit |
| Repayment condition | At maturity | At maturity |  |
| Traveling distance | 0.5 km | 5 km |  |
| Additional credit cost  (e.g. bank charges) | MGA 2,000 | MGA 10,000 |  |
| I will choose … |  |  |  |

| Choice set 5 | | | |
| --- | --- | --- | --- |
| Attribute | Digital credit | Conventional credit | Opt-out |
| Loan duration | 1 month | 12 months | I prefer no |
| Interest amount per month | MGA 12,000 | MGA 12,000 | credit |
| Repayment condition | Instalment | Instalment |  |
| Traveling distance | 1 km | 10 km |  |
| Additional credit cost  (e.g. bank charges) | MGA 2,000 | MGA 14,000 |  |
| I will choose … |  |  |  |

| Choice set 6 | | | |
| --- | --- | --- | --- |
| Attribute | Digital credit | Conventional credit | Opt-out |
| Loan duration | 3 months | 6 months | I prefer no |
| Interest amount per month | MGA 16,000 | MGA 16,000 | credit |
| Repayment condition | At maturity | At maturity |  |
| Traveling distance | 1 km | 5 km |  |
| Additional credit cost  (e.g. bank charges) | MGA 6,000 | MGA 6,000 |  |
| I will choose … |  |  |  |

| Choice set 9 | | | |
| --- | --- | --- | --- |
| Attribute | Digital credit | Conventional credit | Opt-out |
| Loan duration | 3 months | 12 months | I prefer no |
| Interest amount per month | MGA 16,000 | MGA 8,000 | credit |
| Repayment condition | At maturity | At maturity |  |
| Traveling distance | 0.5 km | 20 km |  |
| Additional credit cost  (e.g. bank charges) | MGA 10,000 | MGA 14,000 |  |
| I will choose … |  |  |  |

| Choice set 11 | | | |
| --- | --- | --- | --- |
| Attribute | Digital credit | Conventional credit | Opt-out |
| Loan duration | 6 months | 12 months | I prefer no |
| Interest amount per month | MGA 20,000 | MGA 16,000 | credit |
| Repayment condition | Instalment | At maturity |  |
| Traveling distance | 1 km | 10 km |  |
| Additional credit cost  (e.g. bank charges) | MGA 6,000 | MGA 10,000 |  |
| I will choose … |  |  |  |

| Choice set 12 | | | |
| --- | --- | --- | --- |
| Attribute | Digital credit | Conventional credit | Opt-out |
| Loan duration | 1 month | 3 months | I prefer no |
| Interest amount per month | MGA 16,000 | MGA 12,000 | credit |
| Repayment condition | Instalment | Instalment |  |
| Traveling distance | 0.5 km | 5 km |  |
| Additional credit cost  (e.g. bank charges) | MGA 10,000 | MGA 10,000 |  |
| I will choose … |  |  |  |

**Block 2**

| Choice set 1 | | | |
| --- | --- | --- | --- |
| Attribute | Digital credit | Conventional credit | Opt-out |
| Loan duration | 6 months | 12 months | I prefer no |
| Interest amount per month | MGA 16,000 | MGA 12,000 | credit |
| Repayment condition | Instalment | At maturity |  |
| Traveling distance | 0.5 km | 5 km |  |
| Additional credit cost  (e.g. bank charges) | MGA 6,000 | MGA 14,000 |  |
| I will choose … |  |  |  |

| Choice set 3 | | | |
| --- | --- | --- | --- |
| Attribute | Digital credit | Conventional credit | Opt-out |
| Loan duration | 6 months | 12 months | I prefer no |
| Interest amount per month | MGA 20,000 | MGA 12,000 | credit |
| Repayment condition | Instalment | Instalment |  |
| Traveling distance | 0.5 km | 10 km |  |
| Additional credit cost  (e.g. bank charges) | MGA 10,000 | MGA 6,000 |  |
| I will choose … |  |  |  |

| Choice set 4 | | | |
| --- | --- | --- | --- |
| Attribute | Digital credit | Conventional credit | Opt-out |
| Loan duration | 3 months | 3 months | I prefer no |
| Interest amount per month | MGA 20,000 | MGA 16,000 | credit |
| Repayment condition | At maturity | Instalment |  |
| Traveling distance | 1 km | 20 km |  |
| Additional credit cost  (e.g. bank charges) | MGA 10,000 | MGA 14,000 |  |
| I will choose … |  |  |  |

| Choice set 7 | | | |
| --- | --- | --- | --- |
| Attribute | Digital credit | Conventional credit | Opt-out |
| Loan duration | 3 months | 6 months | I prefer no |
| Interest amount per month | MGA 12,000 | MGA 16,000 | credit |
| Repayment condition | At maturity | Instalment |  |
| Traveling distance | 0.5 km | 20 km |  |
| Additional credit cost  (e.g. bank charges) | MGA 2,000 | MGA 6,000 |  |
| I will choose … |  |  |  |

| Choice set 8 | | | |
| --- | --- | --- | --- |
| Attribute | Digital credit | Conventional credit | Opt-out |
| Loan duration | 1 month | 3 months | I prefer no |
| Interest amount per month | MGA 24,000 | MGA 8,000 | credit |
| Repayment condition | Instalment | At maturity |  |
| Traveling distance | 1 km | 20 km |  |
| Additional credit cost  (e.g. bank charges) | MGA 6,000 | MGA 6,000 |  |
| I will choose … |  |  |  |

| Choice set 10 | | | |
| --- | --- | --- | --- |
| Attribute | Digital credit | Conventional credit | Opt-out |
| Loan duration | 6 months | 6 months | I prefer no |
| Interest amount per month | MGA 24,000 | MGA 8,000 | credit |
| Repayment condition | At maturity | Instalment |  |
| Traveling distance | 1 km | 10 km |  |
| Additional credit cost  (e.g. bank charges) | MGA 2,000 | MGA 10,000 |  |
| I will choose … |  |  |  |
