# Supplementary material for: Farmers’ willingness to pay for digital and conventional credit: Insight from a discrete choice experiment in Madagascar
Source: PLoS One. 2021 Nov 12;16(11):e0257909. doi: 10.1371/journal.pone.0257909 (PMC8589200; doi:10.1371/journal.pone.0257909)
Supplement: S2 Table — (DOCX) [file pone.0257909.s002.docx]

| Table A1: Determinants of farmers’ preference for credit products estimated by the use of a mixed logit model without accounting for socio-economic characteristics of farmers | | |
| --- | --- | --- |
| Variable | Mean coefficient | SD coefficient |
|  | (Standard error) | (Standard error) |
| ***Digital credit*** |  |  |
| Constant | 6.726*** | -1.084*** |
|  | (0.445) | (0.177) |
| Loan duration | 0.130*** | - |
|  | (0.037) |  |
| Interest amount per month | -0.013*** | - |
|  | (0.001) |  |
| Repayment condition (Instalment = 1) ^a)^ | -0.235*** | - |
|  | (0.070) |  |
| Traveling distance | -0.764*** | - |
|  | (0.255) |  |
| Additional credit cost (Withdrawal fees) | -0.012*** | -0.010** |
|  | (0.002) | (0.004) |
| ***Conventional credit*** |  |  |
| Constant | 7.423*** | 1.034*** |
|  | (0.451) | (0.202) |
| Loan duration | 0.006 | 0.110*** |
|  | (0.027) | (0.027) |
| Interest amount per month | -0.020*** | - |
|  | (0.002) |  |
| Repayment condition (Instalment = 1)^a)^ | 0.309*** | 0.794*** |
|  | (0.078) | (0.104) |
| Traveling distance | -0.065*** | - |
|  | (0.012) |  |
| Additional credit cost (Transaction fees) | -0.000 | - |
|  | (0.002) |  |
| Participants/Observations | 420/7,560 |  |
| *Goodness of fit measures* |  |  |
| AIC | 3,151.012 |  |
| BIC | 3,268.833 |  |
| Log likelihood | -1,558.506 |  |
| LR-Statistic (*χ^2^*) (5 d.f.) | 290.170 |  |
| Prob > chi2 | 0.000 |  |
| *Note:* ***, **, and * indicates statistical significance at the 1%, 5%, and 10% levels, respectively. Halton draws = 1,000. SD indicates standard deviation. Only SD coefficients with statistical significance at the 1%, 5%, and 10% levels are shown. The sign of the estimated standard deviations is irrelevant: interpret them as being positive. ^a)^ Indicates effects-coded variable. | | |
