# Supplementary material for: Farmers’ willingness to pay for digital and conventional credit: Insight from a discrete choice experiment in Madagascar
Source: PLoS One. 2021 Nov 12;16(11):e0257909. doi: 10.1371/journal.pone.0257909 (PMC8589200; doi:10.1371/journal.pone.0257909)
Supplement: S3 Table — (DOCX) [file pone.0257909.s003.docx]

| Table 2A: Determinants of farmers’ preference for credit products estimated by the use of a mixed logit model without the attribute “traveling distance” for both credit products | | | | | |
| --- | --- | --- | --- | --- | --- |
| Variable | Mean coefficient | SD coefficient | Mean WTP | Minimum WTP | Maximum WTP |
|  | (Standard error) | (Standard error) | in MGA | in MGA | in MGA |
| ***Digital credit*** |  |  |  |  |  |
| Constant | 2.464** | - | 20,973** | 4,836 | 37,757 |
|  | (1.004) |  |  |  |  |
| Loan duration | 0.086** | ­- | 729*** | 143 | 1,276 |
|  | (0.036) |  |  |  |  |
| Interest amount per month | -0.012*** | - | - | - | - |
|  | (0.001) |  |  |  |  |
| Repayment condition (Instalment = 1)^a)^ | -0.373*** | - | -3,179*** | -4,887 | -1,937 |
|  | (0.068) |  |  |  |  |
| Additional credit cost (Withdrawal fees) | -0.009*** | 0.014*** | -79*** | -122 | -42 |
|  | (0.002) | (0.003) |  |  |  |
| ***Conventional credit*** |  |  |  |  |  |
| Constant | 2.929*** | - | 15,535 | 5,034 | 26,195 |
|  | (1.056) |  |  |  |  |
| Loan duration | -0.012 | 0.129*** | -62 | -337 | 214 |
|  | (0.026) | (0.024) |  |  |  |
| Interest amount per month | -0.019*** | - | - | - | - |
|  | (0.002) |  |  |  |  |
| Repayment condition (Instalment = 1) ^a)^ | 0.217*** | 0.751*** | 1,148 | 385 | 2,004 |
|  | (0.073) | (0.103) |  |  |  |
| Additional credit cost (Transaction fees) | -0.000 | - | -3 | -23 | 18 |
|  | (0.002) |  |  |  |  |
| ***Interaction variables*** |  |  |  |  |  |
| ***Digital credit*** |  |  |  |  |  |
| Constant x Age | -0.025* | - |  |  |  |
|  | (0.015) |  |  |  |  |
| Constant x Education | 0.114** | - |  |  |  |
|  | (0.046) |  |  |  |  |
| Constant x Mobile phone access ^a)^ | 0.738*** | - |  |  |  |
|  | (0.242) |  |  |  |  |
| Constant x Received credit ^a)^ | -0.529** | - |  |  |  |
|  | (0.230) |  |  |  |  |
| Constant x Risk attitude | 0.523*** | - |  |  |  |
|  | (0.110) |  |  |  |  |
| ***Conventional credit*** |  |  |  |  |  |
| Constant x Age | 0.004 | - |  |  |  |
| **Table 2A (continued)** |  |  |  |  |  |
|  | (0.015) |  |  |  |  |
| Constant x Education | 0.081 | - |  |  |  |
|  | (0.047) |  |  |  |  |
| Constant x Mobile phone access ^a)^ | 0.667*** | - |  |  |  |
|  | (0.251) |  |  |  |  |
| Constant x Received credit ^a)^ | -0.646*** | -0.862*** |  |  |  |
|  | (0.234) | (0.254) |  |  |  |
| Constant x Risk attitude | 0.468*** | - |  |  |  |
|  | (0.113) |  |  |  |  |
| Participants/Observations | 420/7,560 |  |  |  |  |
| *Goodness of fit measures* |  |  |  |  |  |
| AIC | 3,122.417 |  |  |  |  |
| BIC | 3,288.752 |  |  |  |  |
| Log likelihood | -1,537.209 |  |  |  |  |
| LR-Statistic (*χ^2^*) (4 d.f.) | 234.140 |  |  |  |  |
| Prob > chi2 | 0.000 |  |  |  |  |
| *Note:* ***, **, and * indicates statistical significance at the 1%, 5% and 10% levels, respectively. For mean WTP estimates, significance level is for the difference in farmers’ mean WTP between digital credit and conventional credit attributes. We report WTP estimates of non-significant attributes for the sake of comparison. All WTP values are in MGA. MGA: Malagasy Ariary. 1 € = MGA 4,150. SD indicates standard deviation. Only SD coefficients with statistical significance at the 1%, 5% and 10% levels are shown. The sign of the estimated standard deviations is irrelevant: interpret them as being positive. ^a)^ Indicates effects-coded variable. Halton draws = 1,000. Krinsky replications = 10,000. | | | | | |
