# Supplementary material for: Farmers’ willingness to pay for digital and conventional credit: Insight from a discrete choice experiment in Madagascar
Source: PLoS One. 2021 Nov 12;16(11):e0257909. doi: 10.1371/journal.pone.0257909 (PMC8589200; doi:10.1371/journal.pone.0257909)
Supplement: S1 Text — (DOCX) [file pone.0257909.s004.docx]

**Instructions for the respondent**

Please imagine that for the upcoming planting season, you would like to purchase seeds and pay workers for your farm. For this, you need MGA 200,000. You are prepared to borrow the total amount (MGA 200,000) from a microfinance bank. You have two loan options for the MGA 200,000. The two loans offer different loan assessment procedures, repayment options, approval speed, and costs (e.g. interest cost, disbursement/withdrawal costs). Before I introduce the two loan options to you, please, let me explain the following credit attributes to you.

1. Loan duration: This is the time frame for the loan (e.g. 3 months).
2. Interest amount per month: This is the amount of money you have to pay per month for using the loan excluding the principal loan amount.
3. Repayment flexibility: This is the how you have to pay the borrowed money to the lender (e.g. bank). You can choose to pay in instalment (multiple payments) or at maturity (one-time payment at the end of the loan duration).
4. Traveling distance: For conventional credit, this is the distance from your house to the nearest formal financial institution (e.g. microfinance bank). For digital credit, this is the distance from your house to the nearest mobile money agent.
5. Additional credit cost (e.g. bank charges): For conventional credit, it is called transaction or loan processing fees. It is the amount of money you have to pay to the bank for processing your loan application. It is paid only once per loan application. For digital credit, it is called withdrawal fees. It is the amount of money you have to pay to the mobile money agent in order to change your digital money to physical money. It is paid every time that you have to change your digital money to physical money.

**Option one (conventional credit):**

You can apply for MGA 200,000 from a microfinance bank which is located in the next town from your place of residence. In order to secure this loan (MGA 200,000), the microfinance bank will conduct a loan assessment with your business data (e.g. income, crop calendar), and you have to provide collateral (e.g. household items or livestock) to the microfinance bank before you can access the loan. Additionally, you have to travel from your place of residence (house) to where the microfinance bank is located to apply for the loan. You may have to wait for some time, up to two weeks before the microfinance bank can take a decision on your loan application. If the microfinance bank decides to approve your loan application, you have to travel to the microfinance bank’s location for the loan disbursement. After the disbursement, you have to visit the microfinance bank several times to make your repayments.

**Option two (digital credit):**

You can access your bank account via a mobile phone. With a mobile phone, you can apply for MGA 200,000 from a microfinance bank (through a mobile application) at any time on any day. For this loan, the loan application and assessment procedure is different from conventional loan. You have to report your business data (e.g. income, crop calendar) through a mobile application (software) of the microfinance bank to apply for the loan. The microfinance bank sends your reported data to a mobile network operator (MNO) to determine your loan eligibility. The MNO uses your non-traditional data (e.g. frequency and amount of mobile phone airtime top-up) to determine your loan eligibility. You do not have to provide collateral (e.g. household items or livestock) to secure the loan. The loan application and lending decision can be completed within seconds (at most 24 hours). You do not have to visit the microfinance bank to apply for this loan. If your loan application is approved, you have to visit a service point (e.g. mobile money agent) which may be located in your village or a nearby town in order to disburse the loan. When it is time for you to repay the loan, you have to go to the service point to make payments on your digital account to repay the loan. You do not have to visit the microfinance bank to repay the loan.

With this knowledge, please consider the terms and conditions of the following credit products and determine which is suitable for you to finance the purchase of seeds and pay workers for your farm. You can also decide not to choose any of the two credit products (opt out/no credit). Enumerator presents the choice sets to the farmer.
